# Virioplankton dynamics are related to eutrophication levels in a tropical urbanized bay

**DOI:** 10.1371/journal.pone.0174653

**Published:** 2017-03-31

**Authors:** Anderson S. Cabral, Mariana M. Lessa, Pedro C. Junger, Fabiano L. Thompson, Rodolfo Paranhos

**Affiliations:** 1 Laboratory of Hydrobiology, Institute of Biology, Federal University of Rio de Janeiro (UFRJ), Rio de Janeiro, Brazil; 2 Graduate Program in Ecology, Institute of Biology, Federal University of Rio de Janeiro (UFRJ), Rio de Janeiro, Brazil; 3 Laboratory of Microbiology, Institute of Biology, Federal University of Rio de Janeiro (UFRJ), Rio de Janeiro, Brazil; 4 Laboratory of Limnology, Department of Ecology, Institute of Biology, Federal University of Rio de Janeiro (UFRJ), Rio de Janeiro, Brazil; National Taiwan Ocean University, TAIWAN

## Abstract

Virioplankton are an important and abundant biological component of marine and freshwater ecosystems. Often overlooked, aquatic viruses play an important role in biogeochemical cycles on a global scale, infecting both autotrophic and heterotrophic microbes. Viral diversity, abundance, and viral interactions at different trophic levels in aqueous environments are not well understood. Tropical ecosystems are less frequently studied than temperate ecosystems, but could provide new insights into how physical and chemical variability can shape or force microbial community changes. In this study, we found high viral abundance values in Guanabara Bay relative to other estuaries around the world. Viral abundance was positively correlated with bacterioplankton abundance and chlorophyll *a* concentrations. Moreover, prokaryotic and viral abundance were positively correlated with eutrophication, especially in surface waters. These results provide novel baseline data on the quantitative distribution of aquatic viruses in tropical estuaries. They also provide new information on a complex and dynamic relationship in which environmental factors influence the abundance of bacterial hosts and consequently their viruses. Guanabara Bay is characterized by spatial and seasonal variations, and the eutrophication process is the most important factor explaining the structuring of virioplankton abundance and distribution in this tropical urbanized bay.

## Introduction

Viruses are the most abundant biological entities on the planet [1–3] and are found in both marine and freshwater ecosystems [4], in and on sediments, in surface waters, and in deep seas [5]. It is currently estimated that global viral abundance (VA) may be as high as 10^31^ particles [5–7] and that viral activity significantly influences ecosystem structuring [8]. Viral lysis affects the composition and diversity of the microbial communities, suggesting that virioplankton are an important component of the microbial food web [7,9–11]. Nutrients are released during viral lysis and thus redistributed into the water column. The consequent transfer of microbial biomass influences nutrient cycling and alters pathways of organic carbon use by prokaryotes [11] in both natural and anthropogenic environments.

Although the importance of virioplankton in structuring microbial communities is now well recognized, several aspects of this phenomenon are not understood. Some coastal regions have been studied in an attempt to understand the effects of human activity on their ecology [12] and the contribution of viral lysis to the structure of microbial communities [13]. For such studies, precise virus counting is critical for a full understanding of viral roles and interactions within microbial communities [1,14]. Typically in aquatic ecosystems, VA is affected by water quality and host abundance [15–21].

Estuaries are considered to be among the most productive ecosystems in the world [12] because they receive large amounts of allochthonous inputs (natural or anthropogenic). In urbanized estuaries, inputs of human sewage usually results in degradation [22,23]. VA in tropical estuarine regions has seldom been studied, and it is unclear how microbial abundance, water quality parameters, and seasonality relate to VA in tropical estuaries. Among tropical coastal bays, Guanabara Bay (GB) is an important ecosystem, but information regarding its virioplankton has not been published. In the present study we provide original data on viral abundance and distribution, and their relationships with eutrophication patterns. The aim of this study is to investigate to following: (i) how VA is influenced by eutrophication in tropical estuaries; and (ii) The relationships between virioplankton and bacterial and algal hosts. In addition we hypothesize that strong eutrophication in GB is the key factor influencing the structure and functioning of its microbial communities.

## Methodology

### Study area

**Fig 1:**
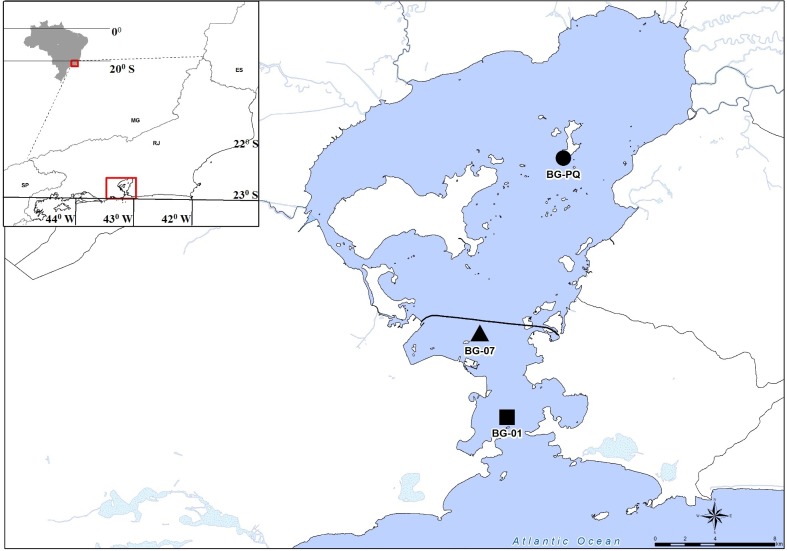
Study site. Guanabara Bay (GB), located in the state of Rio de Janeiro, Brazil. Locations marked as BG-01, BG-07, and BG-PQ indicate the sampling sites within the bay (see text for coordinates).

Guanabara Bay is a tropical estuarine system located in Rio de Janeiro, which is the second-largest city in Brazil (Fig 1). The bay is chronically polluted and is considered one of the most eutrophicated estuaries in the world [24,25] The main human impacts in the bay are related to sewage discharges, oil pollution, garbage disposal, landfills, deforestation, and mangrove forest destruction. Since the impacts are not uniformly distributed, the bay water quality varies spatially, depending on pollution hotspots, tidal influence, and water circulation patterns [24,26]. Dissolved oxygen and salinity levels are higher in the outermost areas of the bay, towards the open ocean, and the inner bay is heavily polluted, with high concentrations of nitrogen and phosphorous compounds, which allow intense phytoplanktonic activity [24]and generally lead to microbial proliferation [27].

### Sampling

Surface and bottom water samples from three sites in GB (22° 50' S, 43° 10' W) were obtained once a month from August 2011 to December 2014. The sampling sites were distributed along the central circulation channel of GB (Fig 1), and were chosen to represent a eutrophication gradient along the bay [24]. Site 01 is located at the entrance to the bay (BG-01: 22° 55' 55" S, 43° 08' 55" W, max depth 35 m), and is affected by ocean water. The second, intermediate, site is located under the Presidente Costa e Silva Bridge (BG-07: 22° 52' 12" S, 43° 09' 41" W, max depth 20 m), which connects Rio de Janeiro city to Niterói city, and the third site, near Paquetá Island (BG-PQ: 22° 46' 18" S, 43° 06' 47" W, max depth 10 m), is affected by river water.

### Flow cytometry counts

Samples for VA assessment were fixed onboard with 0.5% glutaraldehyde, followed by freezing in liquid nitrogen, where they were kept until analysis. Samples for heterotrophic bacterioplankton abundance (BA) assessment were fixed onboard (paraformaldehyde 1% + glutaraldehyde 0.05%), followed by freezing in liquid nitrogen, where they were kept until analysis [28]. Aliquots of both VA and BA samples were stained with SYBR Green I (at a final concentration of 5 × 10^−5^ of the commercial stock solution; Molecular Probes) [29] and analyzed using a FACSCalibur flow cytometer (BD Biosciences) equipped with a 488 nm argon laser. Distinct virus groups and prokaryotic heterotrophic cells with high (HNA) and low (LNA) nucleic acid content weredetected, indentified and quantified based on their signatures in a plot side scatter (X-axis, related by size) versus green fluorescence (Y-axis, green fluorescence from SYBR Green I related to nucleic acid content). The various autotrophic populations were distinguished using a combination of side scatter light and natural fluorescence (red and orange) issued by photosynthetic pigments [30].

Microbial biomass values were calculated based on carbon conversion factors per cell: 0.08 fg per virus [31] and, for BA, 0.20 fg per cell [32]. Chlorophyll *a* (Chl *a*) values were converted to carbon by a factor of 103.9 fg per cell, based on the average conversion factor observed in different parts of the Atlantic Ocean [33].

### Physical, chemical, and biological analyses

Physical, chemical, and biological properties were assessed using standard oceanographic methods [34,35]. Water temperature was measured with a YSI 556 multiparameter system. Salinity, dissolved oxygen (DO), and pH were evaluated using chlorinity, Winkler azide, and potentiometric methods respectively. Chlorophyll *a* analyses were performed after vacuum filtration (< 25 cm of Hg). The filters (cellulose membrane Millipore HAWP 0.45 μm) were extracted overnight in 90% acetone at 4°C and analyzed with a UV-VIS Perkin Elmer Lambda 20 spectrophotometer (Perkin Elmer, USA). Suspended particulate matter determinations were performed by filtration on Millipore AP15 glass-fiber filters. Inorganic nutrients were also analyzed: 1) ammoniacal nitrogen (the sum of N-NH_3_ + N-NH_4_^+^, referred to as ammonia) by indophenol; 2) nitrite by diazotation; 3) nitrate by reduction in a Cd-Cu column followed by diazotation; 4) total nitrogen by digestion with potassium persulfate followed by nitrate determination; 5) orthophosphate by reaction with ascorbic acid; 6) total phosphorous by acid digestion to phosphate; and 7) silicate by reaction with molybdate. Nutrient analyses were performed using a Seal AA3 AutoAnalyzer.

### Statistical analysis

We used the software STATISTICA (StatsoftH) to perform a principal component analysis (PCA) based on a correlation matrix of log_10_-transformed data including total VA, BA, HNA, LNA, Chl *a*, and abiotic variables. All other analyses were performed using the R Statistical Software (version 3.2.2, <www.r-project.org>) [36]. Generalized linear models (GLMs) were used (“glm” function) to test for individual and interactive effects of the two categorical variables, namely sampling station (a three-level fixed factor: BG-01, BG-07, and BG-PQ) and seasonality (a two-level fixed factor: dry and rainy seasons), on the log-transformed response variable VA. All variables (apart from pH) were log_10_-transformed to meet normality (checked using the Shapiro-Wilk test) and homoscedasticity assumptions. Pearson’s correlations were then conducted to verify relationships among the log_10_-transformed variables. We performed a model II linear regression using the major axis method (package “lmodel2” [37,38] between the log-transformed virus biomass and prokaryotic biomass data, and between the virus biomass and phytoplankton biomass data, taken from the whole surface dataset. Slopes and intercepts were compared using the “ma” function (package “smatr”) [39,40], which tests hypotheses about slope or elevation (“elev.test”) based on confidence intervals.

## Results

**Fig 2:**
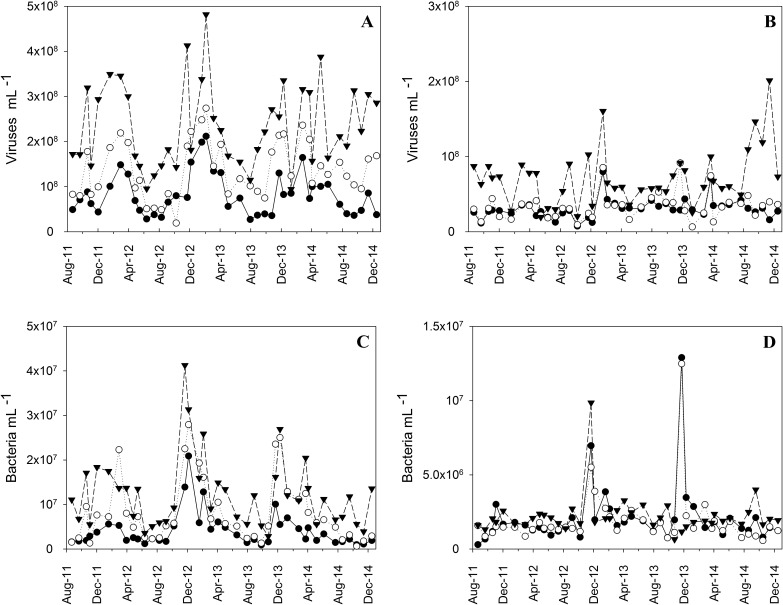
Spatial and temporal distribution of VA and BA at three sites in GB. Note difference in scales between the surface (a, c) and bottom layers (b, d). Symbols are as follows: site BG-01 (full black circle); site BG-07 (empty black circle); site BG-PQ (full black triangle).

**Fig 3:**
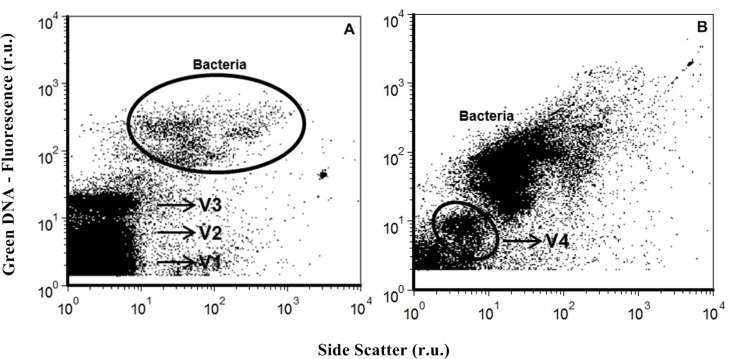
Cytograms showing virus quantification of a sample from the central canal of GB. Enumeration of (a) virus groups V1, V2, and V3, distinguished according to their green fluorescence (V1 had the lowest fluorescence and V3 the highest), and (b) heterotrophic bacteria and virus group V4.

VA recorded in the study area ranged from 0.64 × 10^7^ to 48.18 × 10^7^ viruses mL^-1^. The highest counts were observed at the surface, while the lowest were found in bottom waters (Fig 2A and 2B; Table 1). It was possible to differentiate four different virus groups via flow cytometry (Fig 3A and 3B), as previously described [3,8,41]. They were termed V1, V2, V3, and V4, and were differentiated by their specific increase in fluorescence intensity. Groups V1, V2, and V3 represented, on average, 54%, 32%, and 14% of total VA, respectively. The most abundant group at all depths and sites was V1, which was also the group with the smallest particle sizes (Table 1). All groups exhibited a spatial gradient, with abundance highest in the BG-PQ region (inner bay) and decreasing towards the bay entrance (Fig 2A and 2B; Table 1). Group V4 abundances ranged from undetectable to 0.39 × 10^7^ viruses mL^-1^. This group was mostly observed in surface waters from the innermost site (BG-PQ), although its abundance was generally very low and represented no more than 2% of total VA at this site.

**Table 1:** Microbiological, physical and chemical and water properties (Minimum, maximum, means and standard deviations) at the three sampling sites in GB. Grey rows contain data for surface waters and white rows the data for bottom waters.

			BG-01	BG-07	BG-PQ
Virus Abundance (particles x 10^7^ mL^-1^)	Surface	Min-Max	2.72–21.18	1.88–27.40	9.44–48.18
		Average—SD	8.22 ± 4.67	13.71 ± 6.36	23.59 ±9.36
	Bottom	Min-Max	0.74–7.98	0.64–25.26	2.00–20.12
		Average—SD	3.06 ± 1.35	3.91 ± 3.90	7.20 ± 3.72
V1 Group (particles x 10^7^ mL^-1^)			0.60–15.27	1.11–19.45	4.86–35.05
			4.69 ± 3.41	7.46 ± 4.04	12.41 ± 5.73
			0.38–5.63	0.35–14.97	0.95–10.89
			1.71 ± 0.98	2.17 ± 2.39	3.87 ± 2.19
V2 Group (particles x 10^7^ mL^-1^)			0.77–4.90	0.48–9.46	2.70–25 88
			2.42 ± 1.11	4.14 ± 2.17	7.27 ± 4.64
			0.25–2.11	0.20–9.97	0.59–6.08
			0.97 ± 0.38	1.32 ± 1.49	2.31 ± 1.23
V3 Group (particles x 10^7^ mL^-1^)			0.26 ± 3.38	0 27–5.04	1.13–8.75
			1.09 ± 0.67	2.06 ± 1.32	3.86 ± 2.04
			0.09–0.86	0.08–1.01	0.28–3.66
			0.36 ± 0.15	0.41 ± 0.23	1.01 ± 0.67
Heterotrophic Bacterial abundance			0.91–20.90	0.64–27.94	2.88–41.28
(cell x 10^6^ mL^-1^)			4.11 ± 4.00	8.23 ± 7.33	12.32 ± 7.99
			0.30–12.90	0.57–12.48	0.65–9.86
			2.06 ± 2.06	1.92 ± 1.91	2.24 ± 1.36
Virus-to-Bacteria ratio (VBR)			5.45–77.07	3.30–147.89	5.79–94.39
			27.19 ± 15.35	28.43 ± 26.37	25.07 ± 17.84
			2.23–87.72	4.43–87.22	8.44–114.71
			21.02 ± 15.06	25.04 ± 17.39	37.28 ± 23.77
*Synechococcus* abundance (cell x 10^6^ mL^-1^)			0.00–0.66	0.00–1.19	0.00–2.49
			0.10 ± 0.16	0.21 ± 0.32	0.55 ± 0.63
			0.00–0.13	0.00–0.06	0.00–0.18
			0.04 ± 0.03	0.02 ± 0.02	0.03± 0.04
Chlorophyll *a* (μg. L^-1^)			1.39–66.37	2.14–138.33	6.68–351.50
			16.26 ± 16.25	35.83 ± 32.51	64.89 ± 64.67
			1.07–16.04	0.44–13.90	0.41–13.14
			4.56 ± 3.25	2.77 ± 2.66	2.41 ± 2.37
Water Temperature (°C)			18.00–25.91	20.43–26.86	20.00–27.60
			22.24 ± 1.98	23.32 ± 1.79	23.95 ± 2.02
			15.16–24.44	15.00–25.16	17.55–25.02
			20.21 ± 2.57	20.25 ± 2.80	21.62 ± 1.98
Salinity			28.40–35.18	21.40–34.37	21.42–33.82
			33.62 ± 1.43	31.51 ± 2.77	30.18± 2.86
			33.87–36.00	33.40–35.80	31.92–34.84
			34.92 ± 0.53	34.86 ± 0.54	33.91± 0.65
Dissolved Oxygen (mL.L^-1^)			2.79–7.29	2.42–7.31	1.36–8.27
			4.43 ± 0.89	4.55 ± 1.12	4.40 ± 1.42
			2.10–5.35	2.29–5.18	0.00–4.47
			4.08 ± 0.73	3.59 ± 0.60	2.16 ± 0.91
Total phosphorus (μmol.L^-1^)			0.48–3.18	1.59–7.61	1.81–16.43
			1.84 ± 0.68	3.24 ± 1.33	4.09 ± 2.60
			0.63–1.91	0.64–2.21	1.69–11.23
			1.08 ± 0.27	1.43 ± 0.34	2.74 ± 1.47
Total Nitrogen (μmol.L^-1^)			9.68–71.42	30.78–157.16	15.26–300.01
			39.71 ± 14.20	66.95 ± 22.31	100.39 ± 46.54
			8.23–38.59	12.74–63.91	21.73–217.08
			21.86 ± 6.21	26.55 ± 8.31	47.94 ± 29.24
Total N/Total P			14.96–33.57	12.26–34.10	4.84–43.79
			22.03 ± 3.99	21.56 ± 4.72	26.69 ± 8.69
			8.94–38.30	10.77–54.63	10.63–27.77
			20.79 ± 5.41	19.08 ± 6.70	17.58 ± 3.79
Silicate (μmol.L^-1^)			0.94–37.27	3.63–57.79	5.33–87.00
			11.89 ± 7.20	20.01 ± 11.47	28.82 ± 15.64
			0.07–14.13	1.28–19.50	6.65–38.43
			6.13 ± 2.98	9.00 ± 4.16	15.48 ± 5.88
Transparency (m)			1.00–6.00	0.50–4.50	0.40–2.50
			2.55 ± 1.25	1.73 ± 0.94	1.21 ± 0.49

Seasonal trends were observed in surface waters at all sites (Fig 2A; Table 2). The highest VAs was observed during the summer, and the lowest between May and September (austral winter; Fig 2). This seasonal pattern was independently confirmed through GLM analysis (Table 2), which furthermore confirmed that the effect of seasonality was constant across sampling sites, since there was no interaction between these two factors (Table 2). There was no seasonal effect on VA in bottom waters, however (Fig 2B; Table 2). VAs varied along the estuarine gradient, with highest abundances observed in surface waters close to Paquetá Island (the innermost site) and towards the bay entrance VA decreased (Table 1). With rare exceptions, VA was highest in the inner regions and decreased towards the bay entrance.

**Table 2:** Summary of the generalized linear models (GLMs) of the effects of seasonality (dry and rainy seasons), sampling stations (BG-01, BG-07, and BG-PQ) and their interactions with virus abundance (VA) in GB. Bold *p*-values indicate a statistically significant effect (*p* < 0.05).

Factors	Df	F	p
VA (Surface)			
Stations	2	65.80	**<0.0001**
Seasonality	1	38.98	**<0.0001**
Stations × Seasonality	2	0.82	0.44
Error	121		
VA (Bottom)			
Stations	2	31.14	**<0.0001**
Seasonality	1	0.78	0.38
Stations × Seasonality	2	0.58	0.56
Error	121		

BA ranged from 0.30 × 10^6^ to 41.28 × 10^6^ cells mL^-1^, with highest counts observed at surface waters (Fig 2C and 2D; Table 1). Although less abundant than heterotrophic prokaryotic cells, the cyanobacteria *Synechococcus* exhibited similar distribution patterns at surface waters. As observed for VA, the heterotrophic prokaryotic cells had a stratified distribution along the central channel of the bay. The highest BA (41.28 × 10^6^ cells mL^-1^) was observed in surface waters at the Paquetá Island site (BG-PQ) during the 2012 rainy season, while the minimum value (0.30 × 10^6^ cells mL^-1^) occurred in the bay entrance bottom waters in the 2011 dry season. The same spatial pattern was observed with *Synechococcus*, which decreased towards the coastal waters (Table 1) and had high variability (CV > 220%).

The virus-to-bacteria ratio (VBR) was variable on both spatial and seasonal scales. The mean VBRs increased with the eutrophication gradient: they were higher inside the bay and decreased towards the entrance and cleaner waters (Table 1). There were also seasonal effects: VBRs were higher during the austral winter (June-August), although there were a few exceptions, e.g., an unusually high VBR (42.9) was recorded for the Paquetá Island site bottom waters during summer.

**Fig 4:**
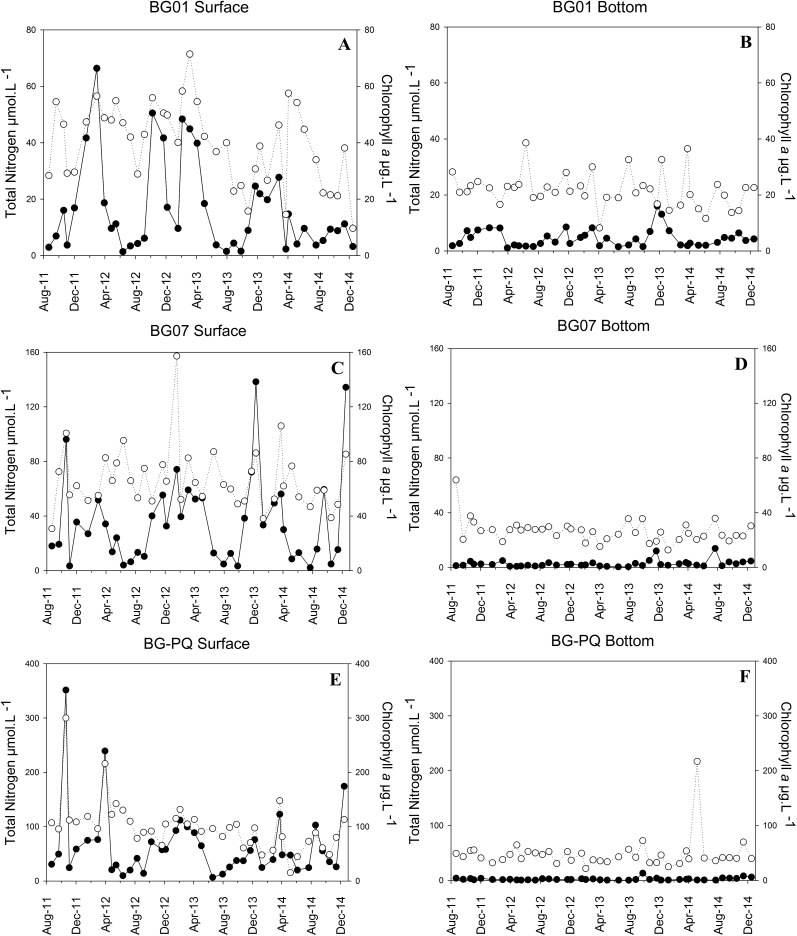
Spatial and temporal distribution of total nitrogen and chlorophyll *a* at the three sampling sites in GB. Note the different scales for the different sites. Symbols are as follows: chlorophyll *a* (full black circle); total nitrogen (empty black circle).

**Fig 5:**
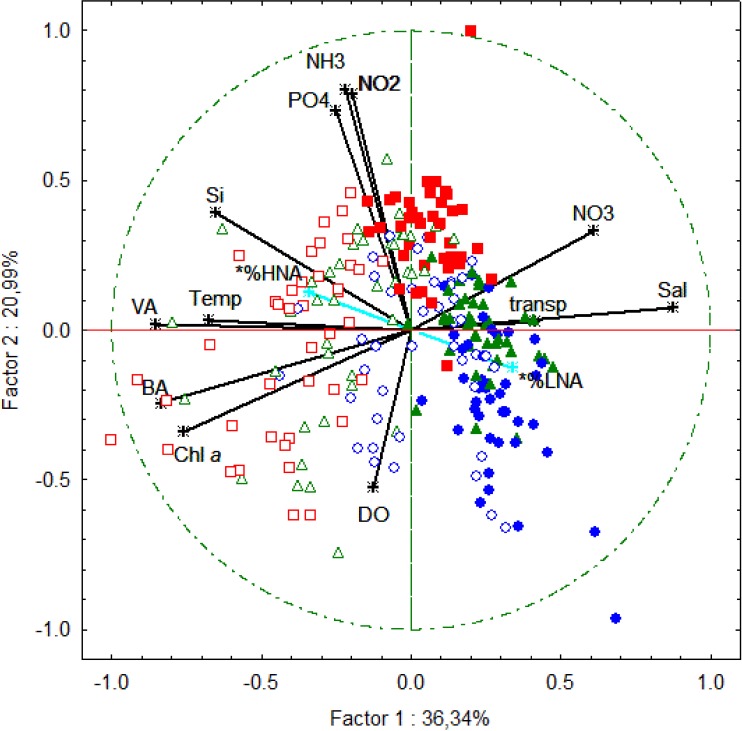
Principal component analysis of the 41 months of sampling in GB. The projection is of the 12 variables and 246 samples. Symbols are as follows: BG-01 surface (empty blue circle); BG-01 bottom (full blue circle); BG-07 surface (empty green triangle); BG-07 bottom (full green triangle); BG-PQ surface (empty red square); BG-PQ bottom (full red square). Temp = temperature; Sal = salinity; DO = dissolved oxygen; TP = total phosphorus; TN = total nitrogen; Sil = silicate; Trans = transparency; Chl *a* = chlorophyll *a*; VA = viral abundance; BA = bacterial abundance; CA = cyanobacterial abundance; %HNA and %LNA: supplementary variables.

The eutrophication gradients were observed for chlorophyll *a* (Fig 4) and for all of the microbial plankton indicators we used from the most heavily enriched or polluted site within the bay, towards the less polluted waters closer to the ocean (Table 1). A PCA was used to integrate the microbiological and chemical data, using a matrix with correlation coefficients from 12 variables and 246 observations. The first two components or factorial axes accounted for 57.3% of total data variability (Fig 5). Factor 1 (PC1) explained 36.3% and was positively correlated with water transparency, salinity, and nitrate, and negatively correlated with phytopigments, temperature, silicate, VA, and BA. This axis (Fig 5) effectively separated the sampling sites according to the water quality gradient, represented by the eutrophicated (negative side) and less-polluted, marine-influenced waters (positive side). Therefore it was considered that axis 1 represents the water quality gradient, suggesting that water quality is the most important factor structuring the VA distribution in the study area. Factor 2 (PC2) explained 21.0% of total data variability, and was correlated negatively with dissolved oxygen, and positively with ammonia, nitrite, and orthophosphate. Samples obtained during the rainy season were mainly distributed along the positive side of this axis (data not shown), whereas those from the dry season were on the negative side. Seasonality was thus considered the second-most important factor structuring VA in GB.

**Fig 6:**
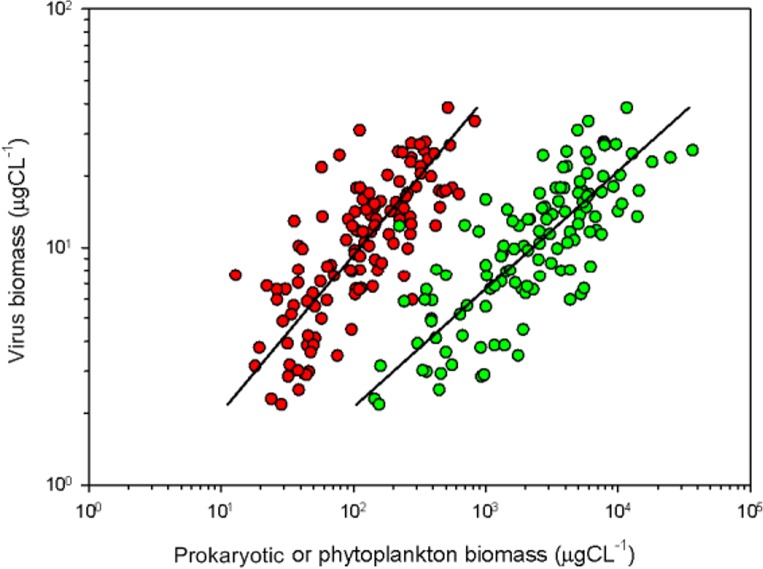
Model II linear regressions between logged viral biomass and logged prokariotic biomass (red circle; r^2^ = 0.59; *p* < 0.0001) and between the log of viral biomass and phytoplankton biomass (green circle; r^2^ = 0.60; *p* < 0.0001) from the surface of the central channel of GB. See Table 4 for confidence intervals.

The VA was correlated (Pearson) to the physical and chemical variables for both the surface and bottom samples (Table 3). For the surface samples, it was positively correlated with total phosphorus, total nitrogen, Chl *a*, and BA, and negatively with salinity. The relationships between VA, prokaryotic cells, and phytoplankton biomass were tested using a Model II linear regression. The steeper slope of the Model II indicates that VA is more dependent on prokaryotic than phytoplanktonic abundance, which reflects the importance of bacteria in host-phage interactions in GB (Fig 6; Table 4).

**Table 3:** Pearson's correlation coefficients between various parameters for the surface waters.

	BA	CA	Temp	Sal	Ph	DO	TP	TN	SiO_4_^-4^	Transp	Chl *a*
VA	0.74***	0.57***	0.50***	-0.56***	0.57***	0.06	0.68***	0.60***	0.43***	-0.64***	0.74***
BA	–	0.75***	0.41***	-0.61***	0.62***	0.18*	0.62***	0.58***	0.48***	-0.69***	0.76***
CA		–	0.42***	-0.45***	0.43***	0.22*	0.36***	0.28**	0.37***	-0.46***	0.50***

VA = viral abundance; BA = heterotrophic bacterioplankton abundance; CA = cyanobacteria abundance; Temp = temperature; Sal = salinity; DO = dissolved oxygen; TP = total phosphorus; TN = total nitrogen; SiO_4_^−4^ = silicate; Trans = transparency; Chl *a* = chlorophyll *a*. Significance of correlations

* *p* < 0.05

** *p* < 0.01

*** *p* < 0.001.

**Table 4:** Slope, intercept and confidence interval (c.i.) of Type II Linear Regression between log Virus biomass vs log bacterial biomass and log phytoplankton biomass.

	Slope	95% [c.i.]	Intercept	95% [c.i.]	*n*	*r*^2^	*p*
VA vs Prokaryotic cells	0.66	[0,57: 0,80]	-0.36	[-0,58:-0,16]	120	0.59	<0.0001
VA vs Phytoplankton	0.50	[0,43: 0,57]	-0.67	[-0,92:-0,43]	120	0.60	<0.0001

## Discussion

In this study, the VA values observed in GB were among the highest reported for estuarine waters (Table 5). Such VA counts are equivalent to Chesapeake and Moreton bays, and higher than those reported for the Yangtze River, Tampa Bay, and the Charente and Bach Dang estuaries. VA data from the global dataset cited indicate that estuaries are favorable environments for high VA, and that these high counts can be attributed to eutrophication and microbial activity in these environments.

**Table 5:** VA in tropical, subtropical, and temperate estuaries. Different methods were used to assess VA: transmission electron microscopy (TEM), epifluorescence microscopy (EFM), and flow cytometry (FCM).

Estuary	Weather	VA (Particles.mL^-1^)	Method	references
Hann Bay—Senegal	Tropical	0.3–2.7 x 10^7^	TEM	[42]
Bach Dang, Red River–Vietnam	Tropical	14.7 x 10^7^	TEM	[43]
Senegal River—Senegal	Tropical	3.8–12.9 x 10^6^	EFM	[22]
Cochin estuary—India	Tropical	1.16–1.98 x 10 ^7^	EFM	[44]
Guanabara Bay—Brazil	Tropical	6.41 x 106–4.82 x 10^8^	FCM	This study
Tampa Bay—USA	Subtropical	4.6 x 10^6^–2.7 x 10^7^	TEM	[45]
Moreton Bay—Australia	Subtropical	0.5 x 10^7^–3.0 x 10^8^	EFM	[18]
Danshui River—Taiwan	Subtropical	3.2–5.0 x 10^7^	EFM	[46]
Chesapeake bay -USA	Subtropical	1.5 x 10^8^	EFM	[12]
Charente Estuary—France	Temperate	6.5–20.8 x 10^7^	EFM	[20]
Yangtze river estuarine, China	Temperate	6.8 x 10^5^−1.7 x 10^7^	FCM	[27]
Mamala Bay—Hawaii	Temperate	Not shown	TEM	[47]

The abundance of all the virus groups followed the same pattern observed for total VA, with highest values reported for the more eutrophic regions of the bay. Group V4 was characterized by a high fluorescence emission. Typically, viruses with high levels of fluorescence emission are associated with algae, and often with eutrophication [8,17]. It is well known that GB is a eutrophic ecosystem [24,25,48], and we found V4 to be most abundant in the most polluted areas, albeit at smaller proportion of the entire virus abundance (max 2%).

Only the surface waters exhibited seasonal changes in virus distribution (Table 1). Indeed, stratification may be more important for structuring virus distribution and abundance in aquatic ecosystems than geographical location [49]. Seasonal temperature changes are less pronounced in tropical estuaries than in temperate estuaries, and there is little difference between summer and winter. Among temperate estuaries, the virus population in Chesapeake Bay (USA) shows clear seasonal trends, despite strong variability across years [12]. However, no seasonal patterns were observed in either the temperate Charente (France) [43] or the tropical Cochin (India) estuaries, and in both these cases there was also no correlation between VA and temperature [44].

It has been reported that eutrophication is the main driver of water quality patterns within GB [24–26]. Our data suggests that eutrophication may also be structuring BA, and consequently VA in this tropical estuary, because it is the main structuring factor identified in the PCA (Fig 5). These findings agree with some VA patterns reported for other estuarine regions globally. Chesapeake Bay there were, surprisingly, no significant differences in VA between sites with different nutrient and salinity concentrations [12], while in Cochin Estuary, VA was explained by bacterial production and correlated with salinity [44]. In the Bach Dang (Vietnam), too, the eutrophication gradient and salinity were considered the main factors shaping picoplankton communities and thus VA [43].

Algae are also potential hosts for viruses; approximately 5% of total VA infects algae [18,20,27,50–52]. Guanabara Bay supports high abundances of several algal groups as a result of eutrophication [24], which results in recurrent algal blooms consisting mostly of flagellated and mixotrophic groups, including potentially harmful species [25]. Despite high phytoplankton numbers, however, the positive correlation of VA with BA and the Chl *a* concentration of surface samples (Table 3) indicates that at the surface, VA was primarily influenced by the distribution of its main hosts. Furthermore, regression results (Fig 6; Table 4) strongly suggest that VA was more dependent on prokaryotic than phytoplanktonic biomass in GB, so we consider BA the main driver of viral abundance and dynamics in the surface waters of the bay. Winget et al [12] hypothesized that virus dynamics in Chesapeake Bay are linked to host abundance, productivity, grazing pressure, and host composition. In addition, it is estimated that viruses are responsible for 10–60% of bacterial mortality, affecting autotrophic and heterotrophic microbial diversity by controlling the abundance of their hosts [2,44,53–55]. There is thus a complex network of causes and effects in such ecosystems, and there are as yet no clear answers regarding which factors control VA in estuaries, however the present results support the hypothesis that host abundance and nutrient concentrations are more important for determining VA than temperature, salinity, or depth, as postulated by several authors [8,18–20].

The ratio between VA and BA has been used to investigate virus-host relationships [2,27,56]. The VBR was high throughout the year, within GB, but was highest during winter, as observed in other estuaries [12,27]. This suggests that host abundance is not the only factor controlling viral dynamics. For example, during the summer months there is more solar radiation, and temperatures are higher, which may be favorable for bacteria and increase their enzymatic activity [27,57], and possibly virus grazing [58], thereby constraining VA during summer. In addition, during summer, there is more rainfall, which results in an increased abundance of suspended particulates that may adsorb viruses [27]. The one exception to this pattern was in the bottom waters from the most eutrophic sampling site (Paquetá Island; data not shown), where VBR was 26% higher during summer. Experimental results regarding nutrient changes [59–61] show that viral production can be increased by alterations in their hosts’ metabolic processes, such as increasing growth rates following nutrient inputs. However, further investigation is necessary to confirm specific virus-host interactions in GB.

We attribute the variability in viral production primarily to virus-to-host interactions, host biomass, and the effect of environmental factors on host populations. Such effects have also been reported for other ecosystems, such as the Charente Estuary, where BA was the most important predictor of VA, explaining approximately 70% of its variability [20]. It is now well known that microbial morphological diversity and life strategies are highly variable, and consequently their associated viral communities are similarly variable [42,62–64]. Our data therefore suggest that eutrophication is the most important factor structuring viral communities in GB and tropical estuaries in general.

## Conclusions

This is the first report on viral abundance in GB (Rio de Janeiro, Brazil), where VA counts are among the highest reported globally. A longer time series could confirm the seasonal patterns observed during the present study; however, our three-year data series suggests that VA in the bay is characterized by spatial and seasonal variations related to environmental conditions and anthropogenic impacts. High correlations between the abundance of viruses and their primary hosts (bacteria) in surface waters, suggests that the high eutrophication in GB is the main driver structuring microbial communities. This study thus provides further indication that eutrophication is a key factor in microbial structuring in tropical estuaries.

## Supporting information

S1 Appendix Virus abundance in Guanabara Bay, RJ—Brazil. Viral abundance of surface and bottom water samples from August 2011 to December 2014.
(XLSX)
